# FDG-PET in the Evaluation of Primary Progressive Aphasia: A Narrative Review

**DOI:** 10.3390/medicina62050800

**Published:** 2026-04-22

**Authors:** Alexandros Giannakis, Emmanouil Anyfantis, Eleni Litsou, Chrissa Sioka, Spyridon Konitsiotis

**Affiliations:** 1 Department of Neurology, Faculty of Medicine, School of Health Sciences, University of Ioannina, Stavrou Niarchou Av., 45500 Ioannina, Greece; skonitso@uoi.gr; 2 Department of Speech Language Therapy, School of Health Sciences, University of Ioannina, Stavrou Niachou Av., 45500 Ioannina, Greece; 3 Department of Otorhinolaryngology, Head and Neck Surgery, University Hospital of Ioannina, Stavrou Niarchou Av., 45500 Ioannina, Greece; 4 Department of Nuclear Medicine, Faculty of Medicine, School of Health Sciences, University of Ioannina, Stavrou Niarchou Av., 45500 Ioannina, Greece

**Keywords:** fluorodeoxyglucose positron emission tomography, primary progressive aphasia, frontotemporal lobar degeneration, Alzheimer’s disease, neuroimaging biomarkers

## Abstract

*Background and Objectives*: Primary progressive aphasia (PPA) and its variants—logopenic (lvPPA), semantic (svPPA), and nonfluent/agrammatic (nfvPPA)—are progressive neurocognitive syndromes characterized by predominant language impairment and associated with heterogeneous underlying neuropathologies. Accurate diagnosis remains challenging due to overlapping clinical features and complex pathobiological mechanisms. Fluorodeoxyglucose positron emission tomography (FDG-PET), which reflects regional cerebral glucose metabolism, may provide valuable insights into both the diagnosis and pathophysiological characterization of PPA. *Materials and Methods*: We reviewed the current literature and identified 48 original research articles that utilized FDG-PET in the evaluation of at least one PPA variant. Eight studies focused exclusively on lvPPA, six on svPPA, and two on nfvPPA, either alone or in comparison with other neurodegenerative diseases. Eighteen studies evaluated at least two PPA variants, while thirteen compared multiple PPA variants with other neurodegenerative disorders. *Results*: Most studies identified characteristic hypometabolic patterns for each PPA variant: left temporoparietal regions in lvPPA, bilateral anterior temporal regions with left predominance in svPPA, and left posterior frontal regions in nfvPPA. These variant-specific metabolic signatures may support differential diagnosis. Additionally, FDG-PET provided important insights into disease progression, including associations with worsening language impairment, evolution toward broader neurodegenerative syndromes, and correlations with specific neurocognitive deficits. These findings are largely consistent with other neuroimaging modalities and disease-specific biomarkers. However, limitations such as small sample sizes and the lack of autopsy confirmation in most studies limit the robustness of the results. *Conclusions*: FDG-PET appears to be a valuable tool for the diagnosis, differential diagnosis, and pathophysiological understanding of PPA. Nevertheless, large-scale, multicenter investigations incorporating pathologically confirmed cases to further validate its clinical utility are needed.

## 1. Introduction

Recent advances in neuroimaging have opened new possibilities for both clinical practice and research in the field of neurocognitive disorders. Brain ^18^F-fluorodeoxyglucose positron emission tomography (FDG-PET) measures cerebral glucose metabolism and can contribute to the diagnosis of cognitive and language disorders associated with different forms of dementia, as well as to the identification of the underlying neuropathological changes accompanying these diseases [1,2]. The European Association of Nuclear Medicine recommends FDG-PET as a diagnostic tool in the evaluation of major neurodegenerative disorders [3].

Primary Progressive Aphasia (PPA) is a neurodegenerative disease and is considered a language-predominant dementia [4]. The core clinical characteristic of PPA is a progressive deterioration of language skills, while non-linguistic cognitive abilities—such as memory, executive function, and visuospatial skills—are relatively preserved in the early stages of the disease [5]. The most frequent language symptoms are word-finding difficulties, impaired sentence processing, and deficits in word comprehension [6]. An accurate diagnosis of PPA requires the integration of clinical, neuropathological, and radiological data [5].

According to current diagnostic criteria, PPA is classified into three distinct clinical subtypes based on clinical presentation. Each subtype is characterized by a specific language profile. Nonfluent/agrammatic variant of PPA (nfvPPA) is characterized by nonfluent, effortful, and groping speech and impaired sentence comprehension. The semantic variant of PPA (svPPA) is characterized by single-word comprehension deficits and semantic impairments. Lastly, the logopenic variant of PPA (lvPPA) presents with word-finding difficulties in confrontation naming tasks and spontaneous speech, along with impaired repetition of phrases and sentences [5]. Brain atrophy associated with PPA predominantly affects the left hemisphere, particularly the anterior temporal lobes and the left perisylvian region [7]. Each subtype of PPA is associated with a specific pattern of atrophy [4].

A comprehensive clinical evaluation of both linguistic and non-linguistic cognitive domains is required for an accurate diagnosis of the disease [8]. In addition to clinical examination, neuroimaging data on the underlying neuropathology are crucial for improving diagnostic accuracy [5]. The purpose of the current narrative review is to examine original research articles that have utilized FDG-PET in the evaluation of PPA and its variants.

## 2. Materials and Methods

We searched the PubMed database from inception through 8 February 2026 using the search algorithm: (“Fluorodeoxyglucose Positron Emission Tomography” OR “FDG-PET”) AND (“primary progressive aphasia” OR “PPA”).

Study selection criteria were as follows:

• Original research articles
• Written in English
• Full text available

Studies were included if they:

• Included at least one patient group that met the clinical diagnostic criteria for PPA (or one or more of its variants), according to the definitions valid at the time of the study, classified as possible or probable
• Included PPA patients who underwent FDG-PET as part of their evaluation.

Given the rarity of PPA and its variants, we did not impose a specific lower limit for sample size. As previously stated, inclusion required that patients fulfil the clinical diagnostic criteria for PPA or its variants. However, when autopsy confirmation was available, this was also reported.

The search yielded a total of 111 articles. One non-English article was excluded. The remaining articles were screened to identify original research studies. A total of 35 meta-analyses, reviews, case reports, case series, and letters to the editor were excluded. Of the 75 original research articles assessed for eligibility, 27 were deemed irrelevant to the study objective (i.e., they did not utilize FDG-PET in the evaluation of at least one patient group with PPA or one of its variants).

**Figure 1:**
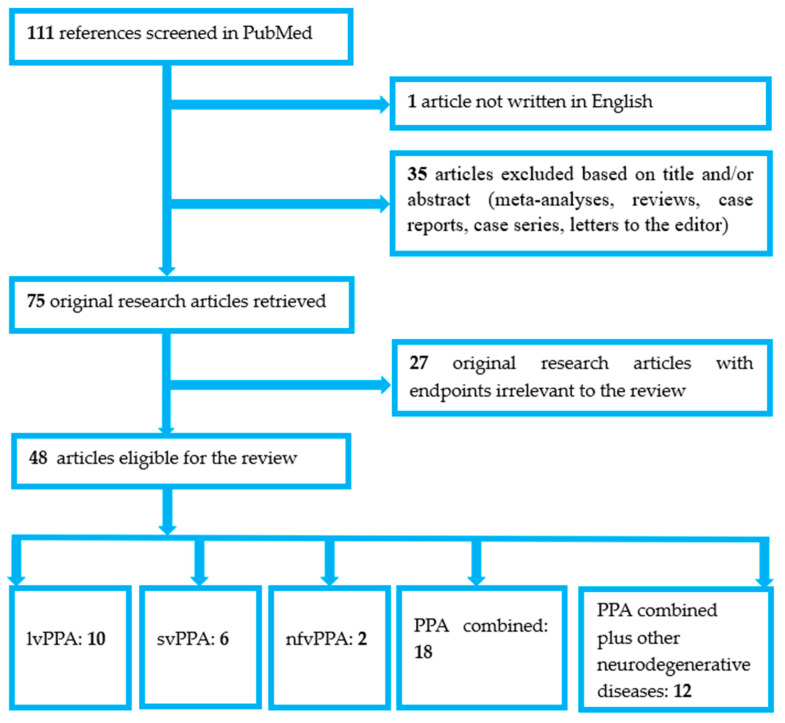
Flow-chart of study selection and categorization.

Ultimately, 48 studies were included in the final analysis. For clarity and improved narrative coherence, the included studies were further categorized into five subgroups: (a) studies including patients with lvPPA only (10 studies); (b) studies including patients with svPPA only (6 studies); (c) studies including patients with nfvPPA only (2 studies); (d) studies including more than one PPA variant or patients with unclassified PPA, but no other neurodegenerative diseases (18 studies); and (e) studies including more than one PPA variant or patients with unclassified PPA and at least one more neurodegenerative disease (12 studies). The study selection process is illustrated in the flow chart shown in Figure 1.

## 3. Results

### 3.1. Studies Enrolling lvPPA Patients

Table 1 summarizes the main findings of studies involving patients with lvPPA.

Whitwell et al. employed multiple imaging modalities, including FDG-PET, Pittsburgh Compound B positron emission tomography (PiB-PET) to assess amyloid-β (Aβ), and magnetic resonance imaging (MRI). They found that patients with high PiB uptake in the occipital lobe exhibited greater hypometabolism in the left parietal lobe on FDG-PET, as well as more pronounced atrophy in the same region on MRI [9]. Subsequently, using the same imaging modalities, they found that patients who were PiB-negative showed asymmetric hypometabolism involving extensive areas of the left hemisphere on FDG-PET, including the inferior temporal gyrus, posterior cingulate cortex, medial prefrontal cortex, temporal pole, hippocampus, caudate nucleus, and fusiform gyrus. In contrast, the PiB-positive group exhibited bilateral hypometabolism on FDG-PET; however, compared with the PiB-negative group, hypometabolism was more pronounced in right hemispheric structures, such as the parietal, temporal, and frontal lobes, as well as the precuneus and putamen. Atrophy patterns on MRI were similar to the aforementioned hypometabolic patterns [10].

Similar patterns to those reported by Whitwell et al. have also been observed in other studies. Madhavan et al. demonstrated that patients with lvPPA showed hypometabolism in the bilateral lateral temporal, lateral parietal, and medial parietal lobes (predominantly left-lateralized), as well as in the left frontal lobe, compared with controls. Interestingly, compared with patients with Alzheimer’s disease (AD), lvPPA patients showed greater hypometabolism in the left lateral temporal lobe (inferior, middle, and superior temporal gyri) and less hypometabolism in the right medial temporal lobe and posterior cingulate cortex. These differences achieved high discriminatory accuracy between the two diseases, particularly when combined with atrophy patterns on volumetric MRI [11]. Likewise, in the study by Matias-Guiu et al., only patients with lvPPA who were amyloid-negative on florbetapir positron emission tomography (Florbetapir-PET) had hypometabolism in the left anterior temporal pole and basal forebrain, whereas the left temporoparietal region was hypometabolic in both amyloid-negative and amyloid-positive individuals [12].

In addition, diverse hypometabolic patterns are observed not only between amyloid-positive and amyloid-negative lvPPA patients but also among amyloid-positive patients themselves. Krishnan et al. grouped lvPPA patients into three arms based on the pattern of amyloid uptake: parietal-predominant, temporal-predominant, and temporoparietal-predominant. More extensive reductions in metabolism were found in the left frontal lobe and precuneus in the first group [13]. Interestingly, amyloid does not appear to be the sole factor underlying the different hypometabolic patterns observed among patients with lvPPA. In a study by Josephs et al., which included only amyloid-negative patients, all patients exhibited hypometabolism in the left temporoparietal region. However, those with progranulin gene mutations were associated with a greater reduction in FGD uptake, extending to the anterior temporoparietal regions [14]. Contrarily, in right-predominant lvPPA, an uncommon syndrome in which atrophy predominantly affects the non-dominant hemisphere, Buciuc et al. found hypometabolism in the non-dominant frontotemporal regions [15]. Underlying pathologies appear to demonstrate distinct metabolic schemes. In a subsequent study by the same group, including autopsy-confirmed cases, patients with lvPPA and pathologically confirmed dementia with Lewy bodies (DLB) showed more marked hypometabolism in the parietal lobe. Those with frontotemporal lobar degeneration (FTLD) exhibited a temporal-predominant pattern, whereas patients with AD pathology demonstrated more heterogeneous patterns. Moreover, compared with patients with clinically probable DLB, those with lvPPA and DLB pathology showed relatively spared metabolism in the non-dominant occipital lobe and more marked bilateral frontal FDG uptake reduction [16]. Additionally, distinct patterns of reduced FDG uptake within the olfactory circuit were demonstrated in patients with lvPPA with underlying AD compared to those with behavioral variant frontotemporal dementia (bvFTD) in a study by Loewenstein et al. [17].

Lastly, FDG-PET has been utilized to investigate correlations between metabolic dysfunction and specific neurocognitive deficits. Giacomucci et al. examined empathy-related impairments in patients with lvPPA and amnestic AD. In the lvPPA group, deficits in perspective-taking were associated with hypometabolism in the left inferior parietal lobule, middle frontal gyrus, insula, and bilateral superior parietal gyri. In contrast, in the amnestic AD group, similar impairments were correlated with reduced metabolism of the right inferior frontal gyrus [18].

In summary, most studies of lvPPA patients report asymmetric hypometabolism predominantly affecting the left hemisphere, with the greatest reductions in FDG uptake observed in the perisylvian temporoparietal cortex. However, this pattern is not universal, as some studies describe bilateral reductions or even right-predominant hypometabolism [10,11,15,18]. With the exception of the studies by Krishnan et al. and Buciuc et al. [13,15,16], most investigations were limited by small sample sizes, which may restrict the generalizability of their findings. Furthermore, only Buciuc et al. included patients with an autopsy-confirmed diagnosis [15,16]. Nevertheless, the majority of studies employed multimodal assessments of PPA, most commonly combining brain MRI and amyloid PET, followed by tau PET, cerebrospinal fluid analysis, and genetic testing. Notably, amyloid PET findings were particularly compelling, as amyloid-positive patients were associated with more pronounced and widespread hypometabolism on FDG-PET [9,10,13]. Nevertheless, other studies did not find more diverse results in amyloid-positive patients [12,14,16].

### 3.2. Studies Enrolling svPPA Patients

The findings of svPPA studies are demonstrated in Table 2.

In the study by Iaccarino et al., cerebral metabolic reduction was observed bilaterally in the anterior temporal poles. Additionally, reduced metabolic activity was detected in the orbitofrontal cortex and several regions of the left temporal lobe, including the inferior, middle, and superior temporal gyri, insula, the inferior fusiform gyrus, and the anterior cingulate cortex. In some cases, structures of the right hemisphere were also involved, including the subiculum and entorhinal [19].

Lu et al. reported similar regional hypometabolism in patients with svPPA, characterized by bilateral involvement of the anterior temporal lobes, with a predominance in the left hemisphere. Reduced metabolic activity was also observed in left-hemispheric regions, including the orbitofrontal gyrus, fusiform gyrus, hippocampus, parahippocampal gyrus, and insula. In contrast, hypermetabolism was detected in other cortical regions, such as the precentral gyrus, postcentral gyrus, lingual gyrus, and calcarine gyrus. This metabolic profile was able to discriminate svPPA from both behavioral variant frontotemporal dementia and Alzheimer’s disease with high diagnostic accuracy. Interestingly, alterations in performance on the Boston Naming Test were correlated with progression of the metabolic pattern in the svPPA group [20]. These alterations appear not only to involve regions typically linked to neurodegeneration in svPPA—such as the anterior temporal pole—but also to affect the functional connectivity of additional, including remote, brain regions. For example, Boccalini et al., using FDG-PET, reported increased functional connectivity in the dorsal parietal cortex, alongside reduced connectivity between temporal and occipital regions compared to healthy controls [21]. Asymmetric hypometabolism of the temporal poles has been shown to correlate with regional atrophy in the same areas, accompanied by additional atrophic changes in several regions of the predominantly affected hemisphere (left or right), as reported by Carlos et al. [22].

Studies other than Lu et al. have also demonstrated associations between hypometabolism, regional atrophy, and specific cognitive deficits in svPPA. For example, Behn et al. reported that word comprehension deficits were correlated with bilateral, left-predominant FDG uptake reduction in the anterior temporal poles. In addition, both word comprehension impairment and regional atrophy were found to correlate with hypometabolism in the same anterior temporal regions, with dysfunction extending posteriorly into the temporal lobe [23]. In contrast, oculomotor behaviors, such as pursuit error and horizontal prosaccade latency, were primarily associated with AD and bvFTD. Reduced FDG uptake was primarily involved in the posterior middle temporal gyrus, whereas it was largely preserved in patients with svPPA. An exception was mildly impaired memory-guided saccades, as reported by Lage et al. [24].

In conclusion, nearly all studies in this subgroup demonstrate bilateral but asymmetrically reduced metabolism of the anterior temporal lobes, with a clear left-sided predominance. In some cases, reduced FDG uptake extends to adjacent regions, including the orbitofrontal cortex and elements of the limbic system [20,21,24]. A major limitation of this subgroup is the absence of autopsy-confirmed cases, along with consistently small sample sizes, as all studies included fewer than 50 patients with svPPA. Brain MRI was the most commonly used adjunctive modality in the evaluation of these patients [19,22,23]. Despite these limitations, several studies reported high diagnostic accuracy in distinguishing svPPA from other neurodegenerative disorders, as well as meaningful correlations between regional atrophy, hypometabolism, and specific neurocognitive deficits [20,23].

### 3.3. Studies Enrolling nfvPPA Patients

FDG-PET studies exclusively focusing on the nfvPPA variant are scarce. Tetzloff et al. compared 11 nfvPPA patients with 20 patients with primary progressive apraxia of speech (PPAOS) and 62 healthy controls. They identified hypometabolism in the inferior, middle, and superior frontal gyri, as well as in the medial temporal lobe, medial and lateral parietal lobes, and the premotor and motor cortices. Additionally, subcortical structures, including the basal ganglia and brainstem, were also hypometabolic. Similar patterns of atrophy were detected on MRI, with diffusion tensor imaging demonstrating dysfunctional fasciculi connecting these regions, such as the superior longitudinal and the right inferior fronto-occipital fasciculus. In contrast, PPAOS patients exhibited more focal dysfunction, primarily involving the motor and premotor cortices. Rates of metabolic decline were greater in the nfvPPA group and correlated with clinical deterioration [25].

Similarly, Sintini et al. utilized FDG-PET, structural MRI, and resting-state MRI to evaluate 18 patients with nfvPPA and 23 patients with PPAOS. They found that alterations in connectivity across multiple regions, rather than dysfunction of a single area, were most strongly correlated with neurodegeneration in both disorders. Specifically, in nfvPPA, the left Broca’s area showed the strongest association with hypometabolism. Furthermore, connectivity to the caudate and thalamus was associated with the rate of metabolic decline and, to a lesser extent, atrophy, in both diseases [26].

### 3.4. Studies of Multiple and Unclassified PPA Subtypes

Table 3 summarizes the main findings of studies involving patients with multiple or unclassified PPA subtypes.

Matias-Guiu et al. used FDG-PET to validate the then recently established diagnostic criteria for PPA [27]. They demonstrated that FDG-PET metabolic activity for each of the three defined PPA variants was highly correlated with the clinical diagnoses based on the criteria proposed by Gorno-Tempini et al. [5]. However, they also found that the lvPPA tended to be overdiagnosed according to the clinical criteria [27]. Subsequently, the researchers investigated whether distinct patterns of hypometabolism across the three variants were associated with the development of non-language neurocognitive deficits. To address this question, they prospectively evaluated patients with PPA using serial FDG-PET imaging. The lvPPA group tended to develop memory impairment, the svPPA group developed behavioral symptoms, and the nfvPPA group showed progression to atypical Parkinsonism, motor neuron disease (MND), and behavioral symptoms. Interestingly, while the hypometabolic patterns in the three variants were largely consistent with findings from previous studies, specific regional associations emerged. In the lvPPA group, left frontal hypometabolism was linked to additional neurocognitive deficits. In contrast, in the nfvPPA group, left anterior temporal hypometabolism was associated with progression to MND, and medial frontal hypometabolism was associated with progression to atypical Parkinsonism [28].

In another study conducted by the same research group, visual versus statistical analysis of FDG-PET images was evaluated across the three PPA variants. Visual assessment demonstrated moderate inter-rater sensitivity and specificity, while statistical analysis achieved higher sensitivity and specificity overall. Notably, poorer agreement between visual analysis and the final diagnosis was observed in the nfvPPA subgroup [29]. Interestingly, they also found that difficulties in reading nonwords (more common in nfvPPA and lvPPA patients) were associated with reduced FDG uptake in the left frontal and temporoparietal cortices. In contrast, left anterior temporal pole dysfunction was more frequently associated with difficulties in reading exception words [30]. Notably, when the researchers attempted to categorize patients across all three clinical PPA variants based on FDG uptake combinations and their longitudinal progression, they identified six distinct PPA subtypes. This classification emerged from subdividing nfvPPA into three groups and lvPPA into two groups according to their specific regional hypometabolism. Importantly, these subgroups demonstrated differing clinical trajectories and showed variable associations with amyloid positivity on molecular imaging, mainly in the lvPPA patients [31].

In the study by Routier et al., patients across the three PPA variants exhibited comparable patterns of cortical hypometabolism. Importantly, MRI revealed white matter alterations corresponding to these hypometabolic regions. In lvPPA, left superior and inferior longitudinal fasciculi, as well as the left inferior fronto-occipital fasciculus, were associated with left temporoparietal hypometabolism. In svPPA, bilateral inferior longitudinal and uncinate fasciculi were linked to bilateral, left-predominant, anterior temporal reduced metabolism. Finally, in nfvPPA, alterations in the left uncinate fasciculus, anterior thalamic radiation, and right superior longitudinal fasciculus were associated with hypometabolism in the left inferior and middle frontal gyri and the supplementary motor area [32]. Similarly, Tau-PET demonstrated diagnostic performance at least comparable to that of FDG-PET, as Josephs et al. observed. Importantly, tau uptake patterns closely mirrored the characteristic hypometabolic patterns previously described for each PPA variant: left temporoparietal involvement in lvPPA, bilateral, left-predominant, anterior temporal involvement in svPPA, and left-predominant prefrontal involvement in nfvPPA [33].

Interestingly, FDG-PET hypometabolism similar to that previously described for each PPA variant has also been observed in patients with PPA-U, as demonstrated in a study by Utianski et al. This finding suggests that even when patients do not fully meet clinical criteria for a specific variant, underlying FDG uptake may still resemble the canonical network signatures of lvPPA, svPPA, or nfvPPA [34]. Similarly, contralateral (right-predominant) patterns of hypometabolism have been observed in a subset of cases. In a study by Ferrari et al., 26% of patients with lvPPA exhibited hypometabolism in the right middle and superior temporal gyri and right supramarginal gyrus. Notably, this subgroup performed better on selected working memory tasks compared with patients showing the more typical left-predominant pattern [35].

Moreover, patients with PPA and probable underlying AD pathology (either lvPPA or PPA-U with mixed logopenic and nonfluent features) who eventually progressed to total aphasia exhibited different metabolic activity. Specifically, Mazzeo et al. showed reduced metabolism in the precuneus, paracentral lobule, and the middle and superior temporal gyri. In contrast, those with a milder disease course demonstrated reduced FDG uptake in the fusiform, middle, and inferior temporal gyri. The first group also had higher CSF tau concentrations [36].

Catricalà et al. utilized FDG-PET in patients with either a PPA variant or PPA-U and found that specific alterations in metabolism in certain brain regions were associated with particular linguistic and other neurocognitive deficits. Specifically, semantic errors were associated with hypometabolism in the left fusiform gyrus, anterior temporal pole, and the middle and inferior temporal gyri. Likewise, syntactic errors were mainly linked to hypometabolism in the left inferior, middle, and superior frontal gyri. Finally, working memory impairment was associated with reduced FDG uptake in the left angular and supramarginal gyri, inferior parietal lobule, posterior cingulate cortex, and the middle and inferior temporal gyri [37]. Similarly, Matías-Guiu et al. investigated whether specific spontaneous speech deficits could discriminate between controls and patients with PPA, as well as among PPA variants. In addition to demonstrating high discriminative ability, they found that lexical deficits correlated with metabolic reductions in the middle and inferior frontal gyri. Fluency deficits were associated with hypometabolism in the left superior and middle frontal gyri and the supplementary motor cortex. Finally, syntactic deficits were linked to reduced metabolic activity in the bilateral superior and middle temporal gyrus, angular gyrus, precuneus, posterior cingulate cortex, and inferior parietal lobule [38]. Similarly, Polito et al. assessed the diagnostic accuracy of specific naming tests in discriminating patients with PPA from controls and among PPA variants. They also found that hypometabolism of the anterior temporal pole and fusiform gyrus was associated with lexical and semantic deficits in the svPPA group [39].

Gómez-Grande et al., in an attempt to validate static first-minute-frame PET imaging, found hypometabolic patterns in PPA variants similar to those reported in the previously mentioned studies [40].

Shir et al. conducted one of the few studies enrolling PPA patients exclusively with an autopsy-confirmed diagnosis. Patients representing all three PPA variants were included. FDG-PET hypometabolic patterns predicted both the clinical diagnosis and the underlying pathology with moderate accuracy and sensitivity, and high specificity. More specifically, the nfvPPA variant was more frequently associated with primary tauopathy and, less commonly, with AD or FTLD–transactive response DNA binding protein 43 kDa (TDP-43) pathology. TDP-43 pathology was most commonly linked to the svPPA clinical phenotype and, to a lesser extent, to lvPPA. In contrast, the lvPPA hypometabolic pattern was most often attributed to underlying AD pathology, although some cases were associated with DLB [41].

Mazzeo et al. studied patients with lvPPA, svPPA, and PPA-U. The latter group exhibited overlapping features of lvPPA and svPPA. While the lvPPA and svPPA groups demonstrated the typical hypometabolic activity described in previous studies, the PPA-U group showed more extensive hypometabolism in the left temporoparietal cortex, involving the anterior temporal pole, inferior and middle temporal gyri, fusiform gyrus, and angular gyrus. Notably, two diverse and equally sized subgroups emerged within the PPA-U group based on AD-specific biomarker positivity [42]. Likewise, Santi et al. evaluated the linguistic deficits of patients with svPPA, lvPPA, and PPA-U using FDG-PET. Semantic deficits in the svPPA group were most commonly associated with reduced FDG uptake of the fusiform gyrus, whereas posterior temporoparietal hypometabolism was predominantly linked to the lexical, phonological, and working memory deficits observed in the lvPPA group. In contrast, the PPA-U group showed more heterogeneous patterns. However, a subgroup of patients characterized primarily by phonological and semantic deficits (referred to as “lvPPA-plus” by the authors) was associated with reduced metabolism of the anterior fusiform and posterior temporal gyri [43]. Lastly, Giacomucci et al. evaluated emotion recognition and empathy deficits in patients with the three PPA variants and examined their association with FDG-PET metabolic patterns. They found that, in the lvPPA group, hypometabolism of the supramarginal gyrus, inferior and middle frontal gyri, middle cingulum, insula, and supplementary motor cortex, predominantly in the left hemisphere, was associated with emotion recognition deficits. In contrast, emotion recognition deficits in the svPPA group were primarily linked to right-hemisphere metabolic reduction, involving the inferior and middle temporal gyri, orbitofrontal cortex, fusiform and parahippocampal gyri, as well as the anterior cingulate cortex. Finally, no correlations were identified between focal metabolic activity and emotion recognition deficits in the nfvPPA group [44].

Overall, the reviewed studies suggest that FDG-PET hypometabolic patterns tend to align with the established clinical variants of PPA, supporting its potential utility as a diagnostic and pathophysiological tool. Characteristic metabolic patterns—typically involving the left temporoparietal regions in lvPPA, anterior temporal lobes (left-predominant) in svPPA, and left frontal regions in nfvPPA—were frequently reported and often showed associations with specific cognitive, linguistic, and behavioral deficits [36,37,44], as well as with white matter changes and, in some cases, underlying molecular pathology [31,32,33]. Longitudinal and multimodal studies further indicated possible links between regional hypometabolism and disease progression, the emergence of non-language symptoms, and biomarker profiles, particularly amyloid and tau deposition [31,32,36]. However, several inconsistencies and limitations were also evident. Considerable overlap between variants, heterogeneity within groups (especially lvPPA and PPA-U), and the identification of additional subtypes based on metabolic patterns complicate straightforward classification [27,28,34,39,40]. Atypical presentations, including right-predominant hypometabolism and mixed clinical phenotypes, were also described [32,35,44]. Moreover, the small sample size and the limited number of autopsy-confirmed cases [41] further constrain the strength of these findings. Overall, while FDG-PET provides useful insights at the group level, its reliability at the individual level remains variable.

### 3.5. Studies Multiple and Unclassified PPA Subtypes Versus Other Neurodegenerative Diseases

The primary findings of studies involving patients with multiple or unclassified PPA subtypes and other neurodegenerative diseases that utilized FDG-PET are summarized in Table 4.

Rabinovici et al. studied three distinct PPA variants and compared them with healthy controls and patients with AD. Compared with AD patients, all three PPA variants demonstrated asymmetrical hypometabolism predominantly affecting the left hemisphere. More specifically, the lvPPA group showed reduced temporoparietal metabolic activity, which was located in the anterior temporal lobe for the svPPA and frontal lobe for nfvPPA groups, respectively [45].

Josephs et al. utilized FDG-PET to examine metabolic patterns in patients who, based on current diagnostic criteria, would be classified as lvPPA, svPPA, nfvPPA, PPA-U, or PPAOS. The first two subgroups exhibited a postrolandic pattern of hypometabolism, whereas the nfvPPA, PPA-U, and PPAOS groups demonstrated prerolandic hypometabolism [46].

Ikeda et al. employed multiple neuroimaging modalities, including structural MRI, metabolic imaging, perfusion imaging, and AD–specific biomarkers, to compare the three PPA variants with patients diagnosed with AD. CSF analysis for AD-specific biomarkers was also performed. Their findings demonstrated distinct patterns across the PPA variants. Patients with lvPPA exhibited reduced FDG uptake in the left temporoparietal region. The svPPA was characterized by reduced FDG uptake in the left anterior temporal pole, whereas nfvPPA showed reduced metabolic activity in the left fronto-insular region. Interestingly, the atrophy patterns observed on MRI and the hypoperfusion patterns detected using ^99m^Tc-ethyl cysteinate dimer single-photon emission computed tomography (^99m^Tc-ECD SPECT) corresponded closely to the regions of reduced metabolism in each respective variant. Furthermore, PiB-PET revealed bilateral amyloid deposition in all lvPPA patients, while no amyloid deposition was detected in the svPPA or nfvPPA groups. In contrast, a patient with early-onset AD demonstrated extensive frontotemporal reduced metabolic activity accompanied by volume loss, hypoperfusion, and amyloid deposition in the same regions, reflecting a more widespread pathological process [47]. Taswell et al. also employed both FDG-PET and PiB-PET, using PiB-PET as the reference standard for identifying underlying AD pathology. In a cohort of patients with clinically diagnosed lvPPA, svPPA, nfvPPA, AD, or corticobasal syndrome (CBS), they evaluated whether focal hypometabolic combinations observed on FDG-PET could predict amyloid positivity as determined by PiB-PET. The authors concluded that FDG-PET patterns predicted underlying amyloid pathology with moderate accuracy (84%), outperforming clinical assessment alone, which achieved an accuracy of 65–67% [48].

Cerami et al. enrolled patients with lvPPA, svPPA, nfvPPA, PPA-U, and slowly progressive anarthria and conducted longitudinal follow-up. In addition to confirming the characteristic hypometabolic patterns of each PPA variant, they identified distinct metabolic signatures within subgroups that predicted subsequent clinical progression. Specifically, bilateral hypometabolism in lvPPA predicted progression to AD. Similarly, bilaterally reduced FDG uptake in the svPPA subgroup was associated with progression to FTD. Finally, lower metabolic activity of the parietal lobe, along with subcortical and brainstem structures, predicted evolution of nfvPPA to either CBS or progressive supranuclear palsy (PSP) [49]. Likewise, Bejanin et al. evaluated patients with svPPA, nfvPPA, and bvFTD using MRI to assess atrophy and FDG-PET to examine hypometabolism, with longitudinal follow-up to investigate disease progression. A significant overlap between hypometabolic and atrophy patterns was observed across all groups; however, metabolic dysfunction demonstrated a more widespread distribution than structural changes. In svPPA, progression extended from the anterior temporal pole at baseline to the orbitofrontal and temporoparietal cortices at follow-up. In nfvPPA, involvement expanded from the supplementary motor cortex to the precentral gyrus and prefrontal cortex. Finally, bvFTD evolved from bilateral prefrontal and temporal cortices to include the parietal cortex and additional structures over time [50].

Nuvoli et al. utilized FDG-PET to discriminate between a group of PPA-U patients and another group comprising patients with aphasic symptoms accompanied by other significant neurocognitive deficits, likely attributable to underlying AD or FTD. Using both qualitative and quantitative visual analyses, they attempted to establish a more specific diagnosis within the two groups; however, the results were modest [51]. Rus et al. also identified a specific pattern of FGD uptake reduction, involving the anterior temporal poles, frontal cortex, anterior and middle cingulum, caudate nucleus, and thalamus. This combination of regions was typically observed in bvFTD patients, but was not detected in other neurodegenerative disorders, including svPPA and nfvPPA, or healthy controls. Notably, the contribution of structural atrophy, as assessed by MRI, to this regional hypometabolism was minimal [52]. Similarly, Franceschi et al. used FDG-PET and MRI to differentiate patients with lvPPA, svPPA, nfvPPA, bvFTD, CBS, and PSP. They identified hypometabolic patterns in the three PPA variants consistent with prior literature, i.e., temporoparietal involvement in lvPPA, anterior temporal in svPPA, and posterior frontal in nfvPPA. These patterns differed from those observed in the other three patient groups and corresponded closely to regional atrophy on MRI. However, only two-thirds of patients with PPA exhibited the previously described left-predominant reduced metabolism, whereas approximately one-third showed right-sided predominance [53]. Moreover, another study by Provost et al. examined whether patients with various neurodegenerative disorders and asymmetric hemispheric hypometabolism on FDG-PET also exhibited contralateral cerebellar hypometabolism, a phenomenon known as crossed cerebellar diaschisis. Interestingly, this finding was observed in 24% of patients, most of whom belonged to the svPPA, lvPPA, and CBS groups [54].

Diaz-Alvarez et al. combined FDG-PET imaging with genetic analysis algorithms to discriminate among AD, bvFTD, and PPA, as well as controls. Their algorithm achieved high diagnostic accuracy in distinguishing PPA from controls [55]. Lastly, Khokhar et al. combined FDG-PET with MRI-based cortical thickness analysis to investigate structural and metabolic alterations in patients with PPA, bvFTD, and MCI. They found more extensive alterations in the PPA group compared with both bvFTD and controls, predominantly involving the frontal and temporal lobes, as well as the anterior cingulum [56].

Overall, the reviewed studies indicate that FDG-PET hypometabolic patterns in PPA generally differ from those observed in AD and other neurodegenerative disorders, while still broadly reflecting the established variant-specific distributions [45,47,53]. Across studies, lvPPA was most often associated with left temporoparietal FDG uptake reduction, svPPA with anterior temporal involvement, and nfvPPA with frontal or fronto-insular changes, although these patterns were described using slightly different regional frameworks, such as pre- versus post-rolandic distinctions [45,46,47,53]. Multimodal imaging studies further showed close correspondence between reduced metabolic activity, structural atrophy, and hypoperfusion, and suggested associations with underlying pathology, particularly amyloid deposition in lvPPA [47,48]. Longitudinal investigations also indicated that specific metabolic patterns may be linked to disease progression and phenotypic evolution, including transitions to AD, FTD, CBS, or PSP [49,50]. However, variability across studies remains notable. Some findings highlighted overlapping or atypical patterns, including right-predominant hypometabolism, mixed phenotypes, and limited diagnostic discrimination in certain subgroups such as PPA-U [51,53]. In addition, while FDG-PET showed moderate accuracy in predicting underlying pathology and outperformed clinical assessment in some cases, its diagnostic performance was not uniformly high [48,51]. Other studies also demonstrated that FDG uptake patterns may extend beyond traditionally defined regions or overlap with those seen in related disorders, such as CBS [54]. Notably, none of the studies in this subgroup included autopsy-confirmed cases, and all but two were characterized by sample sizes of fewer than 50 patients [55,56]. Overall, although FDG-PET contributes useful information for differentiating PPA variants and related conditions, inconsistencies in regional patterns, overlap between syndromes, and variability in diagnostic accuracy limit its reliability at the individual level.

## 4. Discussion

Aphasia is a common clinical feature of several neurodegenerative diseases. lvPPA is now recognized as a distinct clinical subtype strongly associated with underlying AD pathology, which represents its most common neuropathological substrate [57,58]. Synucleinopathies, such as DLB and Parkinson’s disease, may also occasionally present with language deficits [59,60]. Moreover, atypical parkinsonian syndromes, including PSP and corticobasal degeneration (CBD), may manifest with language impairment, sometimes even as a presenting feature, most commonly with nonfluent characteristics. Accordingly, a clinical syndrome compatible with nfvPPA is included in the diagnostic criteria for possible PSP and the proposed phenotypes of CBD [61,62]. In addition, svPPA has been associated with MND, which frequently overlaps with bvFTD, as all of these conditions may represent clinical manifestations of underlying TDP-43 pathology [63]. Furthermore, bvFTD may also represent the initial clinical manifestation of PSP and CBD [61,62]. Taken together, these observations highlight the complex and overlapping clinical spectrum of neurodegenerative disorders associated with PPA. Therefore, PPA may result from a heterogeneous range of underlying pathologies, making accurate clinical diagnosis and prognostication particularly challenging, even for experienced clinicians.

In this context, FDG-PET may serve as a valuable tool in supporting differential diagnosis and improving diagnostic accuracy. The majority of studies reported noteworthy findings across all three variants of PPA. First, nearly all studies that enrolled patients with a specific PPA variant consistently identified a distinct hypometabolic pattern associated with each variant. In particular, reduced FDG uptake in the left temporoparietal region appears to be almost universally present in the lvPPA [9,12,14]. In addition, adjacent regions may also be involved, including the left precuneus, posterior cingulate cortex, superior parietal lobule, and frontal lobe [11,13,18]. Furthermore, more remote regions, such as the contralateral cerebellar hemisphere, may also demonstrate reduced metabolism [54].

Contrarily, the svPPA variant appears to be most commonly associated with low metabolic activity of the anterior temporal pole, which may be bilateral but is typically left-predominant [20,21,23]. Additional regions, such as the orbitofrontal cortex, fusiform gyrus, and medial temporal lobe, may also be affected [20,22]. Furthermore, metabolic alterations may extend to more remote regions, including the contralateral cerebellar hemisphere, as has also been reported in lvPPA [54].

In contrast, the nfvPPA has been associated with more extensive reduced FDG uptake, primarily involving the frontal lobes. Nevertheless, the precise pattern varies among studies and may include the supplementary motor area, frontoinsular cortex, prefrontal cortex, as well as the precentral gyrus and the inferior, middle, and superior frontal gyri [32,46,47,50]. Moreover, even in cases of PPA-U, where no single aphasic feature predominates sufficiently to allow classification into one of the three variants, FDG-PET may offer valuable diagnostic support by revealing a hypometabolic pattern suggestive of a specific variant [34,42,51].

Multiple studies have demonstrated moderate to high diagnostic accuracy in discriminating the three PPA variants from healthy controls, from each other, and from other neurodegenerative diseases, such as AD and bvFTD [11,20,33,41,55]. Notably, reduced radiotracer uptake on FDG-PET generally corresponds to regions of structural atrophy identified on MRI [11,14,20,23,25,47]. However, FDG-PET may also reveal metabolic alterations in regions where structural atrophy is not yet evident [23,50,52], suggesting that metabolic dysfunction may precede detectable anatomical changes. This indicates that FDG-PET may enable earlier detection and more accurate differentiation of PPA variants during the early stages of the disease course. Notably, the hypometabolic patterns observed in PPA appear to correspond with findings from neuroimaging modalities other than MRI, such as perfusion patterns identified with ^99m^Tc-ECD SPECT, possibly reflecting different aspects of the same underlying neurodegenerative process [47].

A key issue in interpreting multimodal neuroimaging findings in PPA is the temporal relationship between functional and structural brain changes. Converging evidence suggests that regional hypometabolism detected by FDG-PET may precede overt cortical atrophy on structural MRI, particularly in the early stages of disease, supporting its potential utility as a sensitive biomarker of incipient neurodegeneration [64,65]. Across PPA variants, neurodegeneration is characterized by a spatial concordance between structural and functional imaging findings, whereby regions of cortical atrophy on MRI correspond closely to areas of hypometabolism on FDG-PET [11,23,26,47]. However, this reflects a spatial, rather than strictly temporal, correspondence, and is not uniformly demonstrated across studies. For instance, in svPPA, structural MRI and FDG-PET may highlight partially distinct patterns of neurodegeneration, suggesting that atrophy and hypometabolism do not always co-localize [66]. Such variability likely arises from a combination of heterogeneous underlying pathobiological processes and methodological differences in neuroimaging acquisition and analysis [7,67,68,69]. Taken together, while FDG-PET demonstrates clear promise for early detection by capturing metabolic dysfunction that may antedate structural decline, the extent to which this sequence generalizes across all PPA variants remains incompletely resolved. A more nuanced understanding of these temporal dynamics is therefore essential for optimizing the clinical application of FDG-PET in early diagnosis and disease monitoring.

More importantly, several studies have demonstrated concordance between the distribution of decreased metabolic activity and specific molecular biomarkers. In particular, in lvPPA, where AD is considered the most common underlying pathology, amyloid PET is positive in the majority of patients. In contrast, amyloid radiotracer uptake is substantially less frequent in the other two variants [24,31]. Notably, the latter and hypometabolism tend to overlap [40]; however, in some cases, more extensive hypometabolism, extending beyond the left temporoparietal region and even involving the contralateral hemisphere, has been observed in amyloid PET–positive lvPPA cases [9,10]. In other cases, markedly different patterns of reduced metabolism were observed in the amyloid-negative group, with ref. [29] possibly suggesting a distinct underlying pathobiological process. Supporting this notion, lvPPA patients with specific genetic mutations and a typical FDG-PET hypometabolic pattern have also been reported to be amyloid-negative [14]. In contrast, the apolipoprotein E ε4 allele was far more common in lvPPA patients who were PiB-PET positive, and this subgroup also tended to exhibit CSF biomarker profiles consistent with AD [47].

Similarly, the combination of FDG-PET with tau-PET has shown promising results. Tau-PET demonstrates ipsilateral uptake in lvPPA, involving the same cerebral hemisphere and the contralateral cerebellar hemisphere, where reduced FDG uptake is observed [15,54]. Interestingly, tau-PET uptake is generally greater in the lvPPA variant and exhibits a distinct distribution in svPPA and nfvPPA, mirroring the regions of decreased metabolic activity on FDG-PET [33]. This suggests that tau-PET may provide at least comparable diagnostic utility, whether used alone or in combination with FDG-PET.

Apart from its role in diagnosis and differential diagnosis, FDG-PET appears to provide valuable insights into disease course and progression to other neurodegenerative syndromes. Specifically, bilaterally reduced FDG uptake is associated with an increased risk of progression from lvPPA to AD, from svPPA to FTD or MND, and from nfvPPA to PSP or CBS [29,49]. Furthermore, evidence suggests that specific brain regions are affected sequentially, following the initial, characteristic hypometabolic regions of each variant. For example, hypometabolism initially observed in the anterior temporal poles in svPPA may subsequently extend to the orbitofrontal and temporoparietal cortices. Similarly, in nfvPPA, metabolic dysfunction may progress from the supplementary motor area to involve the precentral gyrus and prefrontal cortex [50]. Additionally, metabolic decline detected by FDG-PET appears to correlate with clinical deterioration and is also associated with alterations in white matter integrity [25].

Furthermore, FDG-PET may also provide insights into the associations between decreased metabolism and specific clinical symptoms. For example, emotion recognition deficits have been associated with metabolic decrements in the left temporoparietal regions in lvPPA and in the right frontotemporal regions in svPPA, respectively [44]. Likewise, lexical, fluency, exception word reading, non-word reading, syntactic, and semantic deficits also appear to correlate with reduced FDG uptake in specific brain regions characteristic of each PPA variant [30,37,38,39]. Specifically, Polito et al. reported that lexico-semantic processing deficits in svPPA were associated with reduced metabolic activity in temporal regions, including the anterior fusiform gyrus, temporal pole, and posterior fusiform gyrus [39]. In a separate study, Matias-Guiu et al. found that lexical features of spontaneous speech correlated with metabolic activity in left frontal regions, particularly the inferior frontal gyrus, while reduced speech fluency was linked to decreased metabolism in the bilateral frontal lobes. In the same patient cohort, syntactic processing abilities were associated with metabolic activity in the bilateral parieto-temporal junction as well as with structural volumes of the left middle and inferior frontal gyri [38]. More recently, Behn et al. demonstrated that word comprehension deficits in svPPA were correlated with lower FDG uptake in the anterior temporal lobes [23]. Interestingly, patients who eventually progress to total aphasia tend to exhibit different metabolic patterns compared to those who do not [36]. Other deficits, such as impairments in perspective-taking and working memory, also appear to be associated with specific hypometabolic patterns in PPA [18].

However, several limitations are evident in the reviewed studies. First, the sample sizes were generally small. In the overwhelming majority of the included studies, fewer than 100 patients were examined, and it was common for each subgroup to include no more than 10 patients. Notably, PPA is a rare disease, and FDG-PET is not a widely available imaging modality, making the recruitment of larger patient cohorts challenging [70,71]. In addition, differences in the relative rarity of the three PPA variants may have disproportionately affected patient recruitment. For example, a meta-analysis reported that Aβ prevalence is substantially higher in the lvPPA compared to the svPPA and nfvPPA variants [72]. Given that AD is the most common cause of dementia [73], individuals with lvPPA may be more readily identified and recruited into studies than those with the other two variants. Nevertheless, this limitation could be addressed through the development and implementation of multicenter studies using standardized FDG-PET protocols. Such an approach would substantially enhance the robustness of the findings and improve their generalizability.

Another important limitation arises from the heterogeneity of the reported metabolic patterns. Although the majority of studies identified characteristic and relatively distinct regions of hypometabolism corresponding to each PPA variant, several studies reported atypical or divergent findings. For instance, although lvPPA is classically associated with decreased metabolic activity primarily in the left temporoparietal regions, some patients exhibited hypometabolism involving either the left hemisphere (more prominently) or both hemispheres [15,35,53]. Such heterogeneity complicates the interpretation of FDG-PET findings and may limit the reliability of hypometabolic patterns as definitive diagnostic biomarkers in individual patients. Moreover, some regions may overlap between PPA variants or with other neurodegenerative diseases, which can further complicate their differential diagnosis using FDG-PET, as noted in a previous systematic review [74]. Several factors may contribute to this variability. First, the presence of mixed or overlapping neuropathologies—most commonly AD pathology alongside FTD and DLB changes—may lead to atypical or less lateralized patterns of hypometabolism [15,16]. Second, differences in disease stage at the time of imaging may significantly influence metabolic distribution, with earlier stages often showing more focal or asymmetric involvement, and more advanced stages demonstrating bilateral or widespread reductions [20,28,49,50]. Additionally, inter-individual variability in baseline functional brain networks may further modulate the regional expression of neurodegeneration, potentially accounting for right-hemisphere or more diffuse patterns in a subset of patients [75]. Together, these factors highlight the complex and multifactorial nature of metabolic heterogeneity and underscore the need for integrative approaches that consider both biological and methodological sources of variability.

Beyond regional metabolic alterations, the observed changes in functional connectivity and metabolism in remote regions, such as crossed cerebellar diaschisis and hemispheric asymmetry, should be interpreted within the broader framework of interhemispheric network dynamics [54,75,76]. Neurodegenerative processes in primary progressive aphasia are increasingly understood to disrupt large-scale brain networks rather than isolated cortical regions, leading to imbalances in transcallosal connectivity and altered interactions between homologous areas of the two hemispheres [77]. In this context, focal left-hemispheric degeneration, which is typical in language-dominant networks, may induce both reduced functional coupling and compensatory recruitment of contralateral regions [78,79]. Such reorganization may manifest as increased right-hemispheric involvement or more bilateral metabolic patterns, particularly as the disease progresses [77,78,80]. Additionally, remote effects, including cerebellar diaschisis, likely reflect downstream consequences of disrupted cortico-cerebellar and cortico-cortical pathways embedded within these distributed networks [54,75,77]. Furthermore, alterations in brain connectivity may result from environmental factors, some of which may arise during brain development [81]. Integrating these findings within an interhemispheric framework underscores the dynamic interplay between degeneration and compensation, highlighting that metabolic and connectivity changes in PPA arise from complex system-level adaptations rather than purely focal pathology.

Furthermore, the specificity of FDG-PET remains suboptimal, as similar patterns of hypometabolism can be observed across different PPA variants and even in other neurodegenerative disorders, reducing its discriminative power at the individual level [45,53,54,56]. This is further compounded by the considerable overlap between variants, with mixed phenotypes (particularly in PPA-U) challenging the boundaries of current clinical classifications [36,43,46,51]. In addition, atypical presentations, such as right-predominant hypometabolism or more widespread metabolic involvement, further blur these distinctions [35,44,53]. Finally, while FDG-PET provides valuable information on functional impairment, emerging modalities such as tau-PET may offer greater pathophysiological specificity, as early evidence suggests closer correspondence between tau deposition and disease-specific neuroanatomical patterns [15,16,33,54,82,83,84]. Collectively, these factors highlight the need for cautious interpretation of FDG-PET findings and support the integration of multimodal imaging approaches to improve diagnostic accuracy.

Another issue arises from the fact that, while the majority of studies performed a quantitative assessment of FDG-acquired images, with statistical parametric mapping (SPM) being the tool of choice in most cases, others relied on visual assessment. These differing approaches may raise concerns regarding the statistical significance and comparability of results. For example, when Buciuc et al. utilized a specific cut-off value to assess asymmetrical hypometabolism in patients with lvPPA, a lower percentage of patients with a right-predominant lvPPA variant was identified compared to Ferrari et al., who relied solely on visual assessment of asymmetry [15,35]. A possible explanation proposed by Buciuc et al. is that, although a higher proportion of patients exhibited right-dominant hypometabolic asymmetry, many did not reach the predefined cut-off for statistical significance and were therefore classified as having bilaterally reduced metabolic activity [15]. Discrepancies may be even more pronounced in variants with more heterogeneous pathology, such as nfvPPA, where quantitative statistical analysis tends to yield higher agreement and lower inter-rater variability compared to visual assessment [29]. Nevertheless, qualitative visual analysis may still hold clinical value, as demonstrated by Nuvoli et al. [51]. Indeed, while automatically generated brain regional metabolic maps (normalized against control groups) may facilitate the detection of early PPA cases with complex patterns of hypometabolism, visual assessment may be more effective in classifying advanced PPA cases within the AD or FTD spectrum [51].

The most important limitation of the majority of studies is the scarcity of autopsy-confirmed cases. Indeed, only three research articles included patients who underwent post-mortem pathological examination [15,16,41]. In the remaining studies, FDG-PET findings were validated either against the clinical diagnosis or against other, more disease-specific biomarkers, such as amyloid-PET. However, a major challenge in neurodegenerative diseases is the substantial overlap in clinical phenotypes, which frequently leads to misdiagnosis during life and can only be definitively resolved at autopsy [85]. This diagnostic uncertainty is precisely one of the reasons why biomarkers such as FDG-PET are used: to improve diagnostic accuracy.

Furthermore, accumulating evidence suggests that multiple neurodegenerative pathologies may coexist within the same patient, and co-pathology appears to be the rule rather than the exception [86,87,88,89]. Consequently, confirmation of a specific pathology using a disease-specific biomarker does not necessarily establish that it is the sole pathology, or even the predominant one. For example, although AD is considered the primary underlying cause of lvPPA, accounting for approximately 80% of cases, studies with pathological confirmation have demonstrated that DLB may also significantly contribute to this subtype or even represent the leading pathology in a subset of patients [15,16,41]. Similarly, svPPA is most commonly associated with TDP-43 pathology (approximately 72% of cases), but alternative pathologies, including globular glial tauopathy, AD, and Pick’s disease, have also been identified [41]. In nfvPPA, the distribution of underlying pathologies is even more heterogeneous. CBD accounts for approximately 39% of cases, PSP for 32%, Pick’s disease for 18%, and TDP-43 pathology for 11%. Notably, in this cohort, the diagnostic accuracy for predicting the leading pathology using clinical, neuropsychological, and neuroimaging assessment was only modest (75.6%) [41]. Therefore, in order to establish the true diagnostic value and reliability of FDG-PET, further research incorporating larger numbers of autopsy-confirmed cases is essential.

## 5. Conclusions

In conclusion, FDG-PET appears to provide valuable support in the diagnosis and differential diagnosis of PPA variants, both in comparison with healthy controls and in distinguishing between PPA subtypes and other neurodegenerative diseases. Each PPA variant is associated, in the majority of studies, with a characteristic hypometabolic pattern, which may serve as a useful diagnostic signature. Even in cases of PPA-U, specific hypometabolic patterns observed on FDG-PET that are suggestive of lvPPA, svPPA, or nfvPPA may help guide the correct diagnosis. In situations where uncertainty persists despite FDG-PET findings, the use of more disease-specific molecular imaging, such as tau PET and amyloid PET, in combination with FDG-PET hypometabolic patterns may further aid in distinguishing these complex cases. Furthermore, FDG-PET appears to offer important insights into disease progression, including the evolution toward more severe aphasia or other neurological syndromes, as well as its association with specific neurocognitive deficits. These findings are largely consistent with results obtained from other neuroimaging modalities and disease-specific biomarkers.

However, several important limitations must be acknowledged. Most studies included relatively small sample sizes, and, critically, autopsy confirmation was lacking in the majority of cases, limiting the pathological validity and robustness of the conclusions. In addition, the characteristic combinations of hypometabolic regions were not consistently observed across all studies. Therefore, large-scale, multicenter studies incorporating autopsy-confirmed cases are essential to validate the diagnostic utility of FDG-PET and to further clarify its role in the diagnosis, differential diagnosis, and pathophysiological understanding of PPA.

## Figures and Tables

**Table 1:** Main findings of studies exclusively involving patients with lvPPA ^1^.

Study	lvPPA Patients (*n*)	Other Study Groups (*n*)	Other Diagnostic Modalities	Main Findings
Whitwell et al., 2013 [9]	33	NC ^2^ (40)	-MRI ^3^ -PiB-PET ^4^	-greater left parietal hypometabolism in patients with high uptake on PiB-PET
Whitwell et al., 2015 [10]	26	NC (27)	-MRI -PiB-PET	-asymmetric hypometabolism of the left hemisphere in PiB-negative patients -bilateral hypometabolism in PiB-positive patients, more marked in the right hemisphere when compared to PiB-negative patients
Madhavan et al., 2013 [11]	27	AD ^5^ (27), NC (27)	-MRI	-left-lateralized hypometabolism in the bilateral lateral temporal, lateral parietal, and medial parietal lobes, and left frontal lobe, compared with NCs -greater hypometabolism in the left lateral temporal lobe, less hypometabolism in the right medial temporal lobe and posterior cingulate cortex compared with AD -0.89 AUC ^6^ discrimination between lvPPA and AD, when combining atrophy and hypometabolism patterns
Matías-Guiu et al., 2015 [12]	16	NC (16)	-Florbetapir-PET ^7^	-hypometabolism in the left temporoparietal region compared to NC hypometabolism in the left temporal pole and basal forebrain compared to NC for the amyloid-negative group
Krishnan et al., 2016 [13]	53	-	-PiB-PET -MRI	-hypometabolism in the left frontal lobe and precuneus in the parietal-predominant amyloid-positive group
Josephs et al., 2014 [14]	6	NC (unspecified)	-PiB-PET -MRI -genetic analysis	-hypometabolism and atrophy of the left temporoparietal region in all patients, more severe when mutations in the progranulin gene were present
Buciuc et al., 2021 [15]	103	NC (20)	-PiB-PET -tau-PET ^8^ -MRI -autopsy	-greater hypometabolism of the non-dominant frontal and temporal lobes in the right hemisphere–predominant lvPPA patients
Buciuc et al., 2021 [16]	64	DLB ^9^ (15)	-PiB-PET -tau-PET -genetic analysis -autopsy	-parietal lobe hypometabolism in lvPPA patients with DLB pathology -temporal lobe hypometabolism in lvPPA patients with FTLD ^10^ pathology -diverse hypometabolism in lvPPA patients with AD pathology -non-dominant occipital lobe sparing and bilateral frontal lobe hypometabolism in lvPPA patients with confirmed DLB pathology compared to clinically probable DLB patients
Loewenstein et al., 2024 [17]	15	AD (24), bvFTD ^11^ (16), NC (20)	-	-distinct olfactory circuit regions in patients with lvPPA and underlying AD and bvFTD
Giacomucci et al., 2023 [18]	18	AD (38), SCD ^12^ (31)	-amyloid-PET -genetic analysis -CSF ^13^	-perspective taking correlated with metabolism of the left inferior parietal lobule, middle frontal gyrus, insula and bilateral superior parietal gyrus in the lvPPA group -right inferior frontal gyrus hypometabolism correlated with similar dysfunction in the amnestic AD group

^1^ lvPPA: logopenic variant of primary progressive aphasia, ^2^ NC: normal controls, ^3^ MRI: magnetic resonance imaging, ^4^ PiB-PET: Pittsburgh Compound B positron emission tomography, ^5^ AD: Alzheimer’s disease, ^6^ AUC: area under the curve, ^7^ Florbetapir-PET: florbetapir positron emission tomography, ^8^ tau-PET: tau positron emission tomography, ^9^ DLB: dementia with Lewy bodies, ^10^ FTLD: frontotemporal lobar degeneration, ^11^ bvFTD: behavioral variant frontotemporal dementia, ^12^ SCD: subjective cognitive decline, ^13^ CSF: cerebrospinal fluid.

**Table 2:** Main findings of studies exclusively involving patients with svPPA ^1^.

Study	svPPA Patients (*n*)	Other Study Groups (*n*)	Other Diagnostic Modalities	Main Findings
Iaccarino et al., 2015 [19]	10	-	MRI	-bilateral hypometabolism of the temporal poles, left temporal lobe, limbic structures and orbitofrontal cortex
Lu et al., 2021 [20]	15	AD ^2^ (18), bvFTD ^3^ (14), NC ^4^ (15)	-	-hypometabolism in the anterior temporal poles bilaterally (left predominance) -hypometabolism in left hippocampus, parahippocampal gyrus, fusiform gyrus, insula, orbitofrontal gyrus, and striatum -AUC ^5^ 0.98 and 0.91 in distinguishing svPPA from AD and bvFTD, respectively -correlation between hypometabolic pattern and its progression and Boston Naming Test score in the svPPA group
Boccalini et al., 2022 [21]	44	NC (44)	-	-functional alienation of the anterior temporal poles -downregulation of temporal-occipital connectivity -dysfunction of limbic connectivity -increased connectivity of the dorsal parietal cortex
Carlos et al., 2024 [22]	35	NC (15)	MRI ^6^	-asymmetric, bilateral hypometabolism of the anterolateral and medial temporal lobes
Behn et al., 2025 [23]	11	NC (10)	MRI	-asymmetric (left more than right), bilateral hypometabolism of the anterior temporal poles -hypometabolism associated with both atrophy and word comprehension impairment
Lage et al., 2021 [24]	7	AD (33), bvFTD (24), NC (29)	PiB-PET ^7^, CSF ^8^	-dysfunction of the posterior middle temporal gyrus, but not in the svPPA group

^1^ svPPA: semantic variant of primary progressive aphasia, ^2^ AD: Alzheimer’s disease, ^3^ bvFTD: behavioral variant frontotemporal dementia, ^4^ NC: normal controls, ^5^ AUC: area under the curve, ^6^ MRI: magnetic resonance imaging, ^7^ PiB-PET: Pittsburgh Compound B positron emission tomography, ^8^ CSF: cerebrospinal fluid.

**Table 3:** Main findings of studies involving patients with multiple or unclassified PPA ^1^ subtypes.

Study	PPA Patients (*n*)	Other Study Groups (*n*)	Other Diagnostic Modalities	Main Findings
Matias-Guiu et al., 2014 [27]	lvPPA ^2^ (14), svPPA ^3^ (3), nfvPPA ^4^ (12), PPA-U ^5^ (3)	NC ^6^ (12)	-	-high correlation of brain metabolic pattern and clinical diagnosis for all three PPA variants -overdiagnosis of lvPPA variant
Matias-Guiu et al., 2015 [28]	lvPPA (17), svPPA (4), nfvPPA (12), PPA-U (2)	NC (16)	-	-left anterior temporal and medial frontal hypometabolism is associated with nfvPPA progression to MND ^7^ and atypical Parkinsonism, respectively -left temporoparietal and left frontal hypometabolism in the lvPPA group with other cognitive deficits
Matías-Guiu et al., 2015 [29]	lvPPA (19), svPPA (4), nfvPPA (10)	NC (11)	-	-87.8% sensitivity and 89.9%specificity among raters for FDG-PET ^8^ visual analysis, 96.9% sensitivity and 90.9% specificity for statistical analysis -poorer agreement between visual analysis and final diagnosis in nfvPPA
Matías-Guiu et al., 2017 [30]	lvPPA (11), svPPA (5), nfvPPA (19)	NC (13)	-	-left temporoparietal and frontal hypometabolism associated with nonword reading deficits -left anterior temporal hypometabolism associated with reading of exception words deficits
Matias-Guiu et al., 2018 [31]	lvPPA (61), svPPA (15), nfvPPA (46)	NC (28)	Amyloid PET imaging in 43 participants	-6 distinct PPA subtypes based on FDG-PET metabolic patterns -differing clinical trajectories and variable associations with amyloid imaging positivity between subgroups
Routier et al., 2018 [32]	lvPPA (28), svPPA (45), nfvPPA (13)	NC (23)	MRI ^9^	-lvPPA: hypometabolism in the left temporoparietal junction and inferior, middle, and superior temporal gyri -svPPA: bilateral (left-predominant) anterior temporal pole hypometabolism -nfvPPA: left inferior and middle frontal gyrus and supplementary motor area hypometabolism -white matter alterations corresponding to specific hypometabolic regions
Josephs et al., 2018 [33]	lvPPA (14), svPPA (13), nfvPPA (13)	NC (80)	tau-PET ^10^, PiB-PET, MRI	-equal diagnostic utility of tau-PET and FDG-PET -similar tau uptake and hypometabolic patterns among the three PPA variants
Utianski et al., 2019 [34]	PPA-U (15)	-	PiB-PET, MRI	-typical hypometabolic patterns for lvPPA, svPPA, or nfvPPA can be observed in PPA-U patients
Ferrari et al., 2019 [35]	lvPPA (23), svPPA (19), nfvPPA (26)	NC (77)	MRI, amyloid-PET, CSF ^11^, genetic screening (for a patient subset)	-26% of lvPPA patients with right hypometabolic pattern (middle and superior temporal and right supramarginal gyri)
Mazzeo et al., 2022 [36]	lvPPA (21), PPA-U (5),	NC (77)	MRI, CSF, genetic analysis, amyloid-PET (for patient subset)	-different hypometabolic patterns for patients who progressed to total loss of speech
Catricalà et al., 2022 [37]	lvPPA (27), svPPA (21), nfvPPA (10), PPA-U (9)	NC (unspecified)	MRI, amyloid-PET, CSF	-semantic errors: left fusiform, middle and inferior temporal gyri and anterior temporal pole hypometabolism -syntactic errors: left inferior, middle, and superior frontal gyri hypometabolism -working memory: left angular, supramarginal, middle and inferior temporal gyri, inferior parietal lobule, and posterior cingulate cortex hypometabolism
Matias-Guiu et al., 2022 [38]	lvPPA (45), svPPA (11), nfvPPA (31)	NC (31)	MRI	-lexical deficits: middle and inferior frontal gyri hypometabolism -fluency deficits: left superior, middle frontal gyri and supplementary motor cortex hypometabolism -syntactic deficits: bilateral superior, middle temporal and angular gyri, precuneus, posterior cingulate cortex, and inferior parietal lobule hypometabolism
Polito et al., 2023 [39]	lvPPA (20), svPPA (15), nfvPPA (7), PPA-U (10)	NC (unspecified)	MRI, amyloid-PET, CSF (for a patient subset)	-anterior temporal pole and fusiform gyrus hypometabolism was associated with lexical and semantic deficits in the svPPA group
Gómez-Grande et al., 2023 [40]	lvPPA (4), svPPA (1), nfvPPA (9), PPA-U (3)	-	Amyloid-PET	-similar hypometabolic patterns to previous studies for each PPA variant
Shir et al., 2024 [41]	lvPPA (35), svPPA (18), nfvPPA (28)	-	MRI, autopsy	-moderate diagnostic accuracy (75.6%) of underlying pathology and clinical diagnosis with FDG-PET (83.3% sensitivity and 92.6% specificity)
Mazzeo et al., 2024 [42]	lvPPA (23), svPPA (12), PPA-U (20)	NC (77)	MRI, amyloid-PET, CSF, genetic analysis (for a patient subset)	-temporoparietal hypometabolism for lvPPA group -anterior temporal lobe hypometabolism for svPPA group -extensive left temporoparietal hypometabolism for PPA-U group
Santi et al., 2024 [43]	lvPPA (19), svPPA (20), PPA-U (28)	NC (unspecified)	amyloid-PET (for a patient subset)	-fusiform gyrus hypometabolism for svPPA -posterior temporoparietal hypometabolism for lvPPA -anterior fusiform and middle temporal gyri or more heterogenous patterns for PPA-U
Giacomucci et al., 2025 [44]	lvPPA (34), svPPA (11), nfvPPA (11)	NC (34)	MRI, amyloid-PET, CSF (for a patient subset)	-lvPPA: hypometabolism of left temporoparietal regions associated with emotion recognition deficits -svPPA: hypometabolism of right frontotemporal regions associated with emotion recognition deficits -nfvPPA: no correlations between hypometabolism and emotion recognition

^1^ PPA: primary progressive aphasia, ^2^ lvPPA: logopenic variant of PPA, ^3^ svPPA: semantic variant of PPA, ^4^ nfvPPA: nonfluent/agrammatic variant of PPA, ^5^ PPA-U: unclassified PPA, ^6^ NC: normal controls, ^7^ MND: motor neuron disease, ^8^ FDG-PET: ^18^F-fluorodeoxyglucose positron emission tomography, ^9^ MRI: magnetic resonance imaging, ^10^ tau-PET: tau positron emission tomography, ^11^ CSF: cerebrospinal fluid.

**Table 4:** Main findings of studies involving patients with multiple or unclassified PPA ^1^ subtypes versus other neurodegenerative diseases..

Study	PPA Patients (*n*)	Other Study Groups (*n*)	Other Diagnostic Modalities	Main Findings
Rabinovici et al., 2008 [45]	lvPPA ^2^ (4), svPPA ^3^ (5), nfvPPA ^4^ (6)	AD ^5^ (10), NC ^6^ (12)	MRI ^7^, PiB-PET ^8^	-asymmetric, left hypometabolism for all PPA groups compared to AD -left temporoparietal hypometabolism for lvPPA -left anterior hypometabolism temporal for svPPA -left frontal hypometabolism for nfvPPA
Josephs et al., 2010 [46]	lvPPA (6), svPPA (1), nfvPPA (3), PPA-U ^9^ (1) PPAOS ^10^ (1)	-	-	-prerolandic pattern in nfvPPA, PPA-U, and PPAOS -postrolandic pattern in lvPPA and svPPA
Ikeda et al., 2014 [47]	lvPPA (10), svPPA (4), nfvPPA (10)	AD (50)	MRI, PiB-PET, ^99m^Tc-ECD SPECT ^11^, CSF ^12^, genetic analysis (for a patient subset)	-lvPPA: left temporoparietal hypometabolism -svPPA: left anterior temporal pole hypometabolism -nfvPPA: left fronto-insular region hypometabolism -atrophy on MRI and hypoperfusion ^99m^Tc-ECD SPECT similar to the regions of hypometabolism in each respective variant -PiB-PET: bilateral amyloid deposition in all lvPPA patients, but no deposition in the svPPA or nfvPPA groups
Taswell et al., 2015 [48]	lvPPA (19), svPPA (13), nfvPPA (16)	AD (24), CBS ^13^ (14)	PiB-PET	-moderate diagnostic accuracy (84%) of underlying amyloid pathology in patients with PPA or CBS
Cerami et al., 2016 [49]	lvPPA (17), svPPA (11), nfvPPA (19), PPA-U (5)	SPA ^14^ (3)	-	-lvPPA: bilateral hypometabolism predicted progression to AD -svPPA: bilateral hypometabolism predicted progression to FTD ^15^ -nfvPPA: bilateral hypometabolism predicted progression to CBS or PSP ^16^
Bejanin et al., 2020 [50]	svPPA (16), nfvPPA (7)	bvFTD ^17^ (30), NC (43)	MRI	-svPPA: progression from anterior temporal pole to the orbitofrontal and temporoparietal cortices -nfvPPA: progression from supplementary motor cortex to precentral gyrus and prefrontal cortex -bvFTD: progression from bilateral prefrontal and temporal cortices to parietal cortex
Nuvoli et al., 2019 [51]	PPA-U (18)	AD or FTD (17)	MRI	-bilateral (left predominant) hypometabolism of the temporoparietal cortex and limbic system in the PPA-U group -bilateral prefrontal and temporoparietal hypometabolic pattern in the AD/FTD group
Rus et al., 2023 [52]	svPPA (24), nfvPPA (18)	AD (26), bvFTD (111), CJD ^18^ (16), NC (77)	MRI, CSF	-specific hypometabolic pattern typically observed in bvFTD, but not detected in svPPA and nfvPPA
Franceschi et al., 2021 [53]	lvPPA (5), svPPA (11), nfvPPA (2),	bvFTD (16), CBS (12), PSP (5)	MRI	-characteristic hypometabolic patterns for the three PPA groups compared to other patient groups -2/3 of PPA patients exhibited left predominant and 1/3 right predominant hypometabolism
Provost et al., 2021 [54]	lvPPA (14), svPPA (7), nfvPPA (14),	AD (56), bvFTD (23), CBS (11), PSP (6), MCI ^19^ (38), PCA ^20^ (19), other (9)	MRI, PiB-PET, tau-PET ^21^	-most patients with crossed cerebellar diaschisis belonged to the lvPPA, svPPA, or CBS groups
Díaz-Álvarez et al., 2022 [55]	PPA (68)	AD (88), bvFTD (81), NC (39)	genetic analysis	-90–91% diagnostic accuracy for discriminating PPA patients from NC
Khokhar et al., 2025 [56]	PPA (63)	bvFTD (54), MCI (27), NC (43)	MRI	-more extensive alteration in the frontal and temporal lobes and anterior cingulum for PPA compared to bvFTD

^1^ PPA: primary progressive aphasia, ^2^ lvPPA: logopenic variant of PPA, ^3^ svPPA: semantic variant of PPA, ^4^ nfvPPA: nonfluent/agrammatic variant of PPA, ^5^ AD: Alzheimer’s disease, ^6^ NC: normal controls, ^7^ MRI: magnetic resonance imaging, ^8^ PiB-PET: Pittsburgh Compound B positron emission tomography, ^9^ PPA-U: unspecified PPA, ^10^ PPAOS: primary progressive apraxia of speech, ^11 99m^Tc-ECD SPECT: ^99m^Tc-ethyl cysteinate dimer single photon emission computerized tomography, ^12^ CSF: cerebrospinal fluid, ^13^ CBS: corticobasal syndrome, ^14^ SPA: slowly progressive anarthria, ^15^ FTD: frontotemporal dementia, ^16^ PSP: progressive supranuclear palsy, ^17^ bvFTD: behavioral variant FTD, ^18^ CJD: Creutzfeldt-Jakob disease, ^19^ MCI: mild cognitive impairment, ^20^ PCA: posterior cortical atrophy, ^21^ tau-PET: tau positron emission tomography.

## Data Availability

No new data were generated during the preparation of this manuscript.

## Abbreviations

The following abbreviations are used in this manuscript:

Aβ	amyloid beta
AD	Alzheimer’s disease
AUC	area under the curve
bvFTD	behavioral variant frontotemporal dementia
CBD	corticobasal degeneration
CBS	corticobasal syndrome
CJD	Creutzfeldt–Jakob disease
DLB	dementia with Lewy bodies
^18^F-PET	florbetapir positron emission tomography
FDG-PET	^18^F-fluorodeoxyglucose positron emission tomography
FTLD	frontotemporal lobar degeneration
lvPPA	logopenic variant of PPA
MCI	mild cognitive impairment
MND	motor neuron disease
MRI	magnetic resonance imaging
nfvPPA	nonfluent/agrammatic variant of PPA
PiB-PET	Pittsburgh Compound B positron emission tomography
PCA	posterior cortical atrophy
PPA	primary progressive aphasia
PPAOS	primary progressive apraxia of speech
PPA-U	unclassified PPA
PSP	progressive supranuclear palsy
SCD	subjective cognitive decline
SPA	slowly progressive anarthria
svPPA	semantic variant of PPA
tau-PET	tau positron emission tomography
TDP-43	transactive response DNA binding protein 43 kDa
^99m^Tc-ECD SPECT	^99m^Tc-ethyl cysteinate dimer single photon emission computerized tomography
